# Sleep study, respiratory mechanics, chemosensitive response and quality of life in morbidly obese patients undergoing bariatric surgery: a prospective, randomized, controlled trial

**DOI:** 10.1186/1471-2482-11-28

**Published:** 2011-10-17

**Authors:** Luis VF Oliveira, Isabella C Aguiar, Raquel P Hirata, Newton S Faria Junior, Israel S Reis, Luciana MM Sampaio, Claudia S Oliveira, Paulo TC Carvalho, Fernando SS Leitao Filho, Lilian C Giannasi, Lia Azevedo Pinto, Carlos Alberto Malheiros, Wilson Rodrigues Freitas

**Affiliations:** 1 Master's and Doctoral Degree Program in Rehabilitation Sciences, Nove de Julho University, Sao Paulo, Brazil; 2 Sleep Laboratory, Nove de Julho University, Sao Paulo, Brazil; 3 School of Medicine, Fortaleza University, Fortaleza, Ceara, Brazil; 4 Psychology Service, Santa Casa de Misericórdia Hospital, Sao Paulo, Brazil; 5 Surgery Department, Santa Casa de Misericórdia Hospital, Sao Paulo, Brazil

## Abstract

**Background:**

Obesity is a major public health problem in both developed and developing countries alike and leads to a series of changes in respiratory physiology. There is a strong correlation between obesity and cardiopulmonary sleep disorders. Weight loss among such patients leads to a reduction in these alterations in respiratory physiology, but clinical treatment is not effective for a long period of time. Thus, bariatric surgery is a viable option.

**Methods/Design:**

The present study involves patients with morbid obesity (BMI of 40 kg/m^2 ^or 35 kg/m^2 ^to 39.9 kg/m^2 ^with comorbidities), candidates for bariatric surgery, screened at the Santa Casa de Misericórdia Hospital in the city of Sao Paulo (Brazil). The inclusion criteria are grade III morbid obesity, an indication for bariatric surgery, agreement to participate in the study and a signed term of informed consent. The exclusion criteria are BMI above 55 kg/m^2^, clinically significant or unstable mental health concerns, an unrealistic postoperative target weight and/or unrealistic expectations of surgical treatment. Bariatric surgery candidates who meet the inclusion criteria will be referred to Santa Casa de Misericórdia Hospital and will be reviewed again 30, 90 and 360 days following surgery. Data collection will involve patient records, personal data collection, objective assessment of HR, BP, neck circumference, chest and abdomen, collection and analysis of clinical preoperative findings, polysomnography, pulmonary function test and a questionnaire on sleepiness.

**Discussion:**

This paper describes a randomised controlled trial of morbidly obese patients. Polysomnography, respiratory mechanics, chemosensitive response and quality of life will be assessed in patients undergoing or not undergoing bariatric surgery.

**Trial Registration:**

The protocol for this study is registered with the Brazilian Registry of Clinical Trials - ReBEC (RBR-9k9hhv).

## Background

Obesity is currently one of the most serious public health problems. The prevalence of this condition has grown in an accentuated fashion in recent decades, even in developing countries, leading to a global epidemic. Over 1.6 billion adults worldwide are overweight, among which 400 million are obese. The World Health Organisation predicts that 10% of the global population will be obese by 2015 [1]. As poverty has been undergoing a process of eradication, obesity has become a more frequent and more serious problem than malnutrition [2,3]. In adults, overweight is characterised by a body mass index (BMI) between 25 kg/m^2 ^and 29.9 kg/m^2 ^and obesity is defined beginning at 30 kg/m^2 ^[4].

When excess weight reaches very high values (BMI ≥ 40 kg/m^2^), obesity is considered a severe dysfunction due to the association with diseases that are either caused or aggravated by this condition, corresponding to grade III obesity, which is also denominated morbid obesity [4]. The most frequent comorbidities are systemic arterial hypertension [5,6], type II diabetes mellitus [7], obstructive sleep apnoea [8], degenerative joint disease [9], dyslipidemia, coronary disease [10,11], respiratory dysfunction [12] and psychosocial problems [13].

Obesity is the most important risk factor for obstructive sleep apnoea (OSA), especially with fat build-up in the upper portion of the abdomen and the neck region. At least 60 to 70% of patients with OSA are obese [14]. Moreover, the incidence among patients with Grade III obesity is 12-fold to 30-fold greater than that in the general population [15,16]. OSA is characterised by recurrent episodes of partial or complete obstruction of the upper airway during sleep in the presence of ventilatory effort, with a consequent drop in oxyhemoglobin saturation, generating negative intrathoracic pressure and arousals [17]. The gold standard for the diagnosis of OSA is basal nocturnal polysomnography (PSG), which is the simultaneous recording of physiological parameters during a night of sleep, involving the analysis of sleep stages, breathing pattern, cardiovascular function and body movements [18,19].

The association between obesity and respiratory sleep disorders was first described in 1918 by William Osler, who was reminded of a Charles Dickens' characters nicknamed "John, the fat boy", a plethoric snorer and terribly sleepy [20]. The treatment of obesity in these patients has since become a priority [21]. Common diseases such as obesity and hypertension should not be analysed without considering respiratory sleep disorder as a possible causal factor. In recent years, the prevalence of such disorders has grown, affecting 40% of the general population [22,23].

The aim of the treatment of obesity is to improve both health and quality of life through enough of a reduction in body weight to eliminate or improve comorbidities and promote psychological wellbeing [24]. Medical management of obesity is routinely the first line of treatment prescribed by physicians and lay persons alike. Medical therapy often involves a combination of calorie restriction, behaviour modification, increased exercise and pharmacotherapy. Behaviour modification in the treatment regimen may help slow the tendency toward weight regain, but does not prevent it entirely. Unfortunately, the medical treatment of morbid obesity provides minimal sustained weight loss in the majority of patients [25,26]. The fact that morbid obesity remains largely refractory to dietary and medication therapy makes bariatric surgery a viable option [27].

Patients with a BMI of greater than 40 Kg/m^2 ^or 35 to 39.9 Kg/m^2 ^and associated to comorbidities are candidates for bariatric surgery. The most often employed surgical methods are divided into three groups: restrictive, malabsorptive and mixed [28,29]. The most common techniques are Fobi-Capella surgery and Scopinaro's biliopancreatic diversion, which are mixed techniques [30].

This study will sleep study, respiratory mechanics, chemosensitive response and quality of life in morbidly obese patients undergoing bariatric surgery.

### Aims and hypotheses

The aims of this study are to assess the evolution of pulmonary function through spirometry, ventilatory mechanics and breathing tests as well as sleep study parameters and quality of life in subjects suffering from morbid obesity undergoing bariatric surgery and determine a possible correlation between weight loss and these physiological variables. We hypothesise that the weight loss induced by surgical intervention reduces the impact of this disease on sleep quality, cardiovascular consequences and quality of life as well as financial expenditures on treatment.

## Methods/design

### Study design

**Figure 1:**
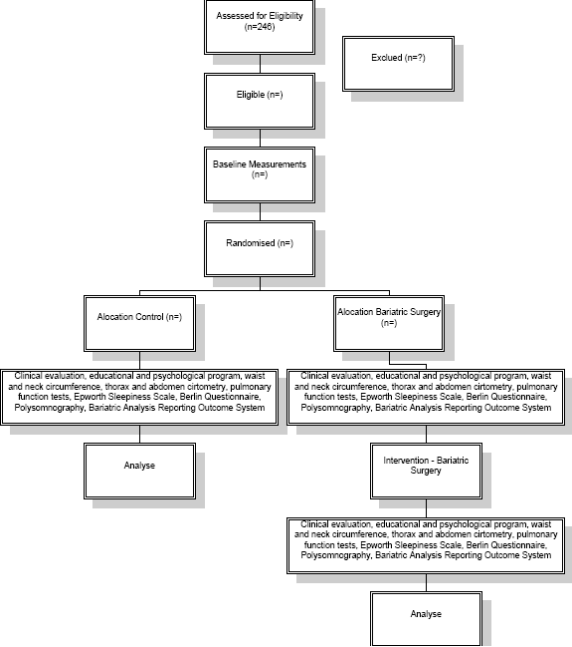
**Flowchart representing the study design**.

The study design is a parallel-group randomised controlled trial and is summarised in Figure 1, and is being conducted according to the ethical standards established in the 1961 Declaration of Helsinki (as revised in Hong Kong in 1989 and in Edimburgh, Scotland in 2000). This study is registered with the World Health Organization Universal Trial Number (UTN) U1111-1121-8873, and *Registro Brasileiro de Ensaios Clínicos *(RBR-9k9hhv), and has been approved by the Human Research Ethics Committees of the Nove de Julho University, São Paulo, Brazil (process number 220506/2009). All participants gave written, informed consent.

### Subjects and recruitment procedure

Individuals with morbid obesity (BMI between 40 kg/m^2 ^and 50 Kg/m^2^) or BMI between 35 kg/m^2 ^and 39.9 kg/m^2 ^associated to comorbidities will be recruited from the surgery ward of the Santa Casa de Misericórdia Hospital of the city of Sao Paulo (Brazil) and sent to the Sleep Laboratory of the *Universidade Nove de Julho*, Sao Paulo (Brazil). Participants will be recruited consecutively and screened for eligibility using a standardised protocol. The eligibility criteria are described below.

### Inclusion Criteria

1- Grade III morbid obesity (BMI ≥ 40 kg/m^2^) or ≥ 35 kg/m^2 ^with comorbidities;

2- Male and female patients aged 18 to 65 years;

3- Documented history of conventional weight loss attempts having proven unsuccessful over time;

4- Agreement to participate in the study through a signed term of informed consent.

### Exclusion Criteria

1- Any medical condition rendering surgery too risky;

2- BMI above 55 kg/m^2^;

3- Clinically significant or unstable mental health concerns;

4- Unrealistic postoperative target weight and/or unrealistic expectations of surgical treatment;

5- Pregnancy, lactation or planned pregnancy within two years of potential surgical treatment;

6- Lack of safe access to abdominal cavity or gastrointestinal tract;

7- Smoking (at least 8 weeks prior to surgery), abusive alcohol use or drug use.

### Randomisation

Following the initial evaluation and fulfilment of the eligibility criteria, the subjects will be randomly distributed into an intervention group [bariatric surgery group (BSG)] and control group (CG). Randomisation numbers will be generated using a randomisation table at a central office. A series of numbered, sealed, opaque envelopes will be used to ensure confidentiality. Each envelope will contain a card stipulating to which group the subject will be allocated. This randomisation criterion will be used due to the enormous number of candidates for bariatric surgery and the limited capacity of the surgical ward to meet this demand. All subjects submitted to this randomisation protocol will meet the eligibility criteria and will be clinically stable.

### Sample size

A previous study published by Lettieri et al. (2008) [31] identified a mean reduction in the Apnoea-Hypnoea Index of 23.4 events/hour in morbidly obese patients submitted to bariatric surgery (expected size effect). Using a standard deviation of 22.8 events/hour from the same study and considering α = 0.05 and power = 80%, the sample was estimated as 17 patients.

### Study interventions

#### Evaluation

All evaluations will occur prior to the surgical intervention. The patients submitted to bariatric surgery will be evaluated again 30, 90 and 360 days following the procedure. All subjects in both groups will be submitted to the following evaluation protocol.

#### Clinical evaluation

General physical measurements and PSG will be performed a week prior to preparation for the surgery by a well-trained physician and physical therapist following recommended procedures and using precise instruments and will include body weight (kg), height (m), calculation of body mass index (BMI) using the formula weight/height^2 ^[32], circumferences (cm) of the neck, waist, and hip [33], heart and respiratory rates, blood pressure values, Mallampati index [34], tonsil index [35], the administration of specific questionnaires for sleep apnoea and excessive daytime sleepiness [36] and a quality of life questionnaire [37].

#### Educational and psychological program

During the initial evaluations, all patients will take part in an educational program in the auditorium of Santa Casa de Misericordia Hospital, where they will receive information on the development and progression of obesity, treatment (pharmacological and non-pharmacological) and the importance of regular physical activity practice. All patients will receive a chart containing the educational program [38].

#### Waist and neck circumference

The measurements of waist and neck circumference will be performed with a metric tape (7 mm in width). The sites of the measurements will be standardized. Waist circumference will be measured at the mid point between the lower edge of the last rib and the iliac crest. Neck circumference will be measured horizontally over the cricoid cartilage [39].

#### Thorax and abdomen cirtometry

Cervical thoracoabdominal cirtometry will be carried out to assess thorax and abdomen mobility and define the diaphragm index. The measurement will be performed by fixing the zero point on the metric tape to the anterior region of the thorax at the level that is being measured (axillary, xiphoid or abdominal), with the tape enveloping the entire thorax or abdomen with maximal possible pressure and the other extremity of the tape placed over this same fixed point. The aim of the maximal possible pressure of the tape on the body is to avoid the interference of soft structures in the measurements. Mobility and range of motion are provided by maximal inspiration and expiration [40].

#### Pulmonary function tests

##### Spirometry

The lung function tests will be carried out with the patient seated in a comfortable position using the KoKo PFT System Version 4.11 (nSpire Health, Inc; Louisville, CO, USA) and following guidelines for the execution of pulmonary function tests of the Brazilian Society of Pneumology [41] and the European Respiratory Society [42]. The subjects will perform the test seated as comfortably as possible, with the body erect and the upper limbs unsupported. All exams will be carried out by a technician trained in obtaining the necessary cooperation from the subjects and appropriately operating the equipment in order to ensure accurate, reproducible results. The equipment will be calibrated prior to each exam with a 3-L syringe [41].

##### Analysis of respiratory mechanics

Maximal inspiratory pressure (MIP) and maximal expiratory pressure (MEP) physiologically constitute the most adequate test for the determination of respiratory muscle strength. MIP is an indicator of ventilatory capacity and the development of respiratory failure and is indicated for the assessment of the degree of abnormality and monitoring of the weakening of inspiratory muscles individually in the progress of patients [43]. The tests will occur on the same day in which the patients undergo spirometry. The tests will be performed in a quiet setting. The patients will be seated comfortably, with the trunk at a 90-degree angle in relation to the thighs and breathing calmly and at rest [41].

##### Rebreathing test

In this study protocol, we will employ the re-inhalation method described by Read [44]. This is a simple technique that has been validated in the literature and allows the determination of the ventilatory response to CO_2 _stimulus. The patients will breathe through a mouthpiece and, after achieving a steady state, basal ventilation will be recorded for three minutes. The patients will then breathe connected to a 15-L latex balloon filled with a 7% CO_2 _and 93% O_2 _mixture for five minutes or until an end-tidal CO_2 _pressure (PetCO_2_) of 70 mm Hg is reached or until exhaustion. Data will be collected using a spirometer. The slope of ventilatory response to carbon dioxide will be obtained from a linear regression between ventilation and PetCO_2_. The data will be adjusted by a 0.9 correction factor to correct flow readings for hyperoxic gas mixture. The rebreathing tests will be carried out at Santa Casa de Misericordia Hospital (Sao Paulo, Brazil). All manoeuvres will be carried out by physicians.

##### Epworth Sleepiness Scale

The Epworth Sleep Scale is a simple, self-administered questionnaire with items addressing situations of daily activities and the occurrence of daytime sleepiness in adults. The subjects will be instructed to classify their likelihood of feeling the desire to nap or sleep in eight situations on a scale of 0 to 3 (0 = no chance of napping; 1 = small chancing of napping; 2 = moderate chance of napping; and 3 = strong chance of napping) [45,46].

##### Berlin Questionnaire

The Berlin Questionnaire is used to identify patients at high risk for sleep disordered breathing in a variety of populations. This clinical history questionnaire has recognized efficacy in distinguishing individuals at greater risk for OSA, with ten items organized into three categories: snoring and apnoea (5 items), daytime sleepiness (4 items), systemic arterial hypertension and obesity (1 item). Any marked response is considered positive. The score is divided into categories. Category 1 is considered positive when there are two or more positive responses to Items 1 to 5. Category 2 is considered positive when there are two or more positive responses to Items 6 to 8. Category 3 is considered positive when the response to Item 9 is "yes" or at BMI equal to or greater than 30 kg/m^2^. Two or more positive categories indicate high risk [47].

##### Polysomnography

A complete full-night PSG will be performed using a digital system (Embla, A10 version 3.1.2 Flaga, Hs. Medical Devices, Iceland) at the Sleep Laboratory of *Universidade Nove de Julho*. All recording sensors will be attached to the patient in a non-invasive manner using tape or elastic bands. The following physiological variables will be monitored simultaneously and continuously: four channels for the electroencephalogram (EEG) (C3-A2, C4-A1, O1-A2, O2-A1), two channels for the electrooculogram (EOG) (EOG-Left-A2, EOG-Right-A1), four channels for the surface electromyogram (muscles of the submentonian region, anterior tibialis muscle, masseter region and seventh intercostal space), one channel for an electrocardiogram (derivation V1 modified), airflow detection via two channels through a thermocouple (one channel) and nasal pressure (one channel), respiratory effort of the thorax (one channel) and the abdomen (one channel) via x-trace belts, snoring (one channel) and body position (one channel) via EMBLA sensors, and arterial oxygen saturation (SaO2) and pulse rate via an EMBLA oximeter. All PSGs will be performed and sleep stages visually scored according to standardized criteria for investigating sleep [48,49]. EEG arousals, sleep-related respiratory events and leg movements will be scored in accordance with the criteria established by the American Academy of Sleep Medicine Manual for Scoring Sleep and Associated Events [50].

The patients will be instructed to remain as relaxed as possible and sleep naturally, as if at home. All signals will be recorded continuously. Throughout the night, the subjects will be monitored by a technician experienced in polysomnography [49].

#### Bariatric surgery

Bariatric surgery will be performed on the subjects selected for the procedure at the Santa Casa de Misericórdia Hospital by experienced specialists. The surgical techniques employed for the treatment of obesity are divided into restrictive, malabsorptive and mixed. The aim of restrictive techniques (vertical gastroplasty with banding or Mason's surgery, adjustable gastric band) is to reduce gastric capacity in order to promote early satiety, thereby reducing the volume of food ingested. The aim of malabsorptive techniques (jejunoileal bypass or Payne's operation) is to reduce the absorption of foods through the exclusion of a segment of small intestine. Mixed techniques [Fobi-Capella surgery, Scopinaro's biliopancreatic diversion, and a Scopinaro technique modified by Marceau and Biron (duodenal-switch)] combine mechanical restriction to the food bolus and intestinal malabsorption [51,52].

#### Bariatric Analysis and Reporting Outcome System (Baros)

The Bariatric Analysis and Reporting Outcome System (BAROS) introduced by Oria and Moorhead in 1997 [53] provides a standard for comparisons of surgical treatment in patients with morbid obesity. This system adds or subtracts points in the evaluation of three main areas (percentage of excess weight loss, changes in medical conditions and assessment of quality-of-life), considering four outcome groups (failure, fair, good and excellent). Points are deducted for complications and reoperative surgery. The comorbidities taken into account by BAROS are hypertension, cardiovascular disease, dyslipidemia, type 2 diabetes, sleep apnoea syndrome, osteoarthritis and infertility. Idiopathic intracranial hypertension, lower extremity venous stasis disease, gastroesophageal reflux and urinary stress incontinence become comorbidities when these conditions impair or diminish quality of life or require long-tem treatment [54].

### Quality control

In order to ensure data quality, the physiotherapists and physicians in charge of the data acquisition in this study will receive specific training. Periodic external monitoring will be performed to verify the adequate application of the methodology in collecting information and performing the different examinations. The results of the preoperative and postoperative exams will be analysed by blinded evaluators.

### Statistical analysis

Data will be presented as means ± standard deviation, when applicable. For comparison of continuous variables before and after bariatric surgery, it will be used the paired Student t-test or Wilcoxon tests as appropriate. Comparisons between groups will be performed using Student *t *test or Mann-Whitney U according to the distribution.

All tests will be 2 tailed, and p values of less than 0.05 will be assumed to represent statistical significance. Multivariate linear regression will be used to identify independent predictors of the AHI following surgical weight loss. All analyses will be done using SPSS ver. 16.0 [55].

## Discussion

Obesity is a chronic and often a life-long disease. It is a major cause of preventable death and is associated with a range of negative physiological and psychological consequences [56]. The aim of this study is to analyse clinical parameters referring to anthropometric data, pulmonary function tests, respiratory mechanics, sleep study, sleepiness scale and chemosensitive response to CO_2 _in patients with morbid obesity submitted to bariatric surgery. If successful, this project will help reduce the negative health, economic and social consequences of obesity.

## Competing interests

The authors declare that they have no competing interests.

## Authors' contributions

All the authors contributed to the conception and design the study. LVFO and ICA provided the idea for the study, established the hypothesis and wrote the original proposal. ICA and LVFO significantly contributed to writing this paper, while LCG, LMMS, LAP, CSO, FSSLF, RPH, ISR, CAM, WRFJ and NSFJ were involved in critically revising the manuscript. This protocol paper was written by ICA and LVFO with input from all co-authors. All authors read and approved the final manuscript.

## Pre-publication history

The pre-publication history for this paper can be accessed here:

http://www.biomedcentral.com/1471-2482/11/28/prepub
